# Hepatoprotective effect of pinostrobin against thioacetamide-induced liver cirrhosis in rats

**DOI:** 10.1016/j.sjbs.2022.103506

**Published:** 2022-11-17

**Authors:** Suhayla H. Shareef, Morteta H. Al-Medhtiy, Ahmed S. Al Rashdi, Peshawa Y. Aziz, Mahmood A. Abdulla

**Affiliations:** a Department of Biology, College of Education, Salahaddin University-Erbil, Erbil, Kurdistan Region, Iraq; b Department of Anatomy and Histology, Faculty of Veterinary Medicine, University of Kufa, Iraq; c Central Public Health Laboratories, Ministry of Health, Oman; d Department of Medical Laboratory Science, Technical College of Applied Science, Sulaimani Polytechnic University, Sulaymaniyah, Kurdistan Region, Iraq; e Department of Medical Microbiology, College of Science, Cihan University-Erbil, Erbil, Kurdistan Region, Iraq

**Keywords:** Pinostrobin, Liver cirrhosis, TAA, Histology, PCNA, TNF-α, IL-6, IL-10, Liver function tests

## Abstract

The study *in vivo* assessed the effect of pinostrobin on the histology, immunohistochemistry, and biochemical parameters of thioacetamide (TAA) induced liver cirrhosis in Sprague Dawley rats. The rats were noticeably gavaged with two doses of pinostrobin (30 mg/kg and 60 mg/kg) with TAA and exhibited a substantial decrease in the liver index and hepatocyte propagation with much minor cell injury. These groups meaningfully down-regulated the proliferation of cellular nucleus antigen (PCNA) and alpha-smooth muscle actin (α-SMA). The liver homogenate displayed augmented antioxidant enzymes, superoxide dismutase (SOD) and catalase (CAT) activities escorted with reducing in malondialdehyde (MDA) level. The serum level of bilirubin, total protein, albumin, and liver enzymes (ALP, ALT, and AST) returned to normal and was similar to that of normal control and silymarin with TAA-treated groups. pinostrobin-fed groups also decreased the level of Tumor necrosis factor-alpha (TNF-α), Interleukin-6 (IL-6), and increased the level of Interleukin-10 (IL-10). Acute toxicity with a higher dose of 500 mg/kg of pinostrobin did not manifest any toxicological signs in rats. The hepatoprotective effect of pinostrobin could be due to potentially inhibited the progression of liver cirrhosis, down-regulation of PCNA and α-SMA proliferation, prevented oxidation of hepatocytes, improved SOD and CAT enzymes, condensed MDA, repairs of liver biomarkers, reduced cellular inflammation and modulation of inflammatory cytokines.

## 1 Introduction

Pinostrobin is a phytochemical compound found naturally in numerous medicinal plants, including Lauraceae, Zingiberaceae, Fabaceae, and Polygonaceae (Dzoyem et al., 2017, Vasas et al., 2020). Pinostrobin is known to have a spectrum of pharmacological effects (Patel et al., 2016), together with antiulcer (Sun et al., 2020), antiplatelet, anti-mutagenic (Zhang et al., 2017), radical scavenging or anti-oxidant, anti-inflammatory, anti-cancer (Jaudan et al., 2018, Sopanaporn et al., 2020, Jones and Gehler, 2020, Sun et al., 2020), antimicrobial (Hernández Tasco et al., 2020), anti-parasitic (Vechi et al., 2020) and gastric curative action (Kanchanapiboon et al., 2020). Although the liver is a highly vital organ for cleansing, liver diseases can be the greatest health complication (Sivakrishnan and Pharm, 2019). Cirrhosis, hepatocellular carcinoma, viral hepatitis, and alcoholic hepatitis are the most common liver diseases, all of which are closely accompanied by jaundice (Rodniem et al., 2018). Numerous medicinal plants are known to protect the liver against the hepatotoxic effects of TAA in experimental animals. These effects are frequently revised in the scientific literature (Amin et al., 2013, Kadir et al., 2014, Azab and Albasha, 2018, Salama et al., 2018, Mousa et al., 2019, Khalil et al., 2021, Shareef et al., 2022, Al-Medhtiy et al., 2022). The most widespread hepatoprotection agent is silymarin, which is an herbal substance extracted from seeds of the *Silybum marinum* plant (Gillessen and Schmidt, 2020). The latter is used broadly as a therapeutic additive to reduce liver disease symptoms such as hepatitis, fatty acid infiltration, and cirrhosis resulting from toxic chemical and alcohol effects (Gillessen and Schmidt, 2020). Several researchers have used silymarin as a reference therapy for hepatoprotection against TAA hepatotoxicity (Rouhollahi et al., 2015, Bagherniya et al., 2018, Yang et al., 2019, Abood et al., 2020, Singh et al., 2020).

TAA increases oxidative stress and attracts free radicals, which causes damage to proteins, lipids, and DNA (da Silva et al., 2021, Elnfarawy et al., 2021). TAA makes hepatic cells impaired after its breakdown into sulphene and sulphone, which is caused by a hazardous path that includes Bio-transformation involving the CYP4502E1 enzyme (Correia and Kwon, 2020). Several studies by different co-researchers evidenced TAA has been used in the early stages of liver fibrosis (Rouhollahi et al., 2015, El-Baz et al., 2019, Urrutia-Hernández et al., 2019, Abood et al., 2020, Jantararussamee et al., 2021). The effectiveness of pinostrobin since traditional rights necessity verified to aid progress novel medicines functioning in contradiction of liver syndromes. However, the hepatoprotective activity of pinostrobin has not been reported previously in an experimental animal study. This study aims to assess the hepatoprotective action of pinostrobin on TAA-induced liver injuries in rats.

## 2 Material and methods

### 2.1 Thioacetamide

TAA was obtained from Sigma-Aldrich, Switzerland, and then liquefied in 10 % Tween 20 and mixed well until completely dissolved. At that time, 200 mg/kg body mass was inserted i.p rat three times weekly for 8 weeks. TAA-induced changes in biological morphology structures comparable to human liver cirrhosis (Kadir et al., 2013).

### 2.2 Silymarin

Silymarin is a reference drug (International Laboratory, USA) used in research as standard medicine. Silymarin was melted in 10 % Tween 20 and then gavaged to rats in a dose of 50 mg/kg (Alkiyumi et al., 2012, Amin et al., 2012).

### 2.3 Pinostrobin

Pinostrobin was purchased from Sigma-Aldrich Chemical Co., (USA). Pinostrobin was dissolved in 10 % Tween 20 and given to rats in doses of 30 and 60 mg/kg (5 mL/kg) (Abdulaziz Bardi et al., 2013).

### 2.4 Acute toxicity study and experimental animals

Thirty-six (18 males and 18 females) healthy Sprague Dawley rats (6–7 weeks old, weigh between 180 and 200 g) were acquired from the Experimental Animal House, Cihan University-Erbil. The rats were given standard rat pellets diet and tap water ad libitum and located in separate cages with a wide-mesh wire bottom to prevent coprophagia. The rats were kept in cages for one week for adaptation. The acute toxicity study was used to determine the safety of pinostrobin. The rats were allocated similarly into 3 groups; vehicle (10 % Tween 20, 5 mL/kg), 250 mg/kg, and 500 mg/kg of the pinostrobin (5 mL/kg). Before the dosing, the rats were fasted overnight (food but not water). Food was withdrawn for a further 3 to 4 h after dosing. The animals were observed for 24–48 h after the administration of the pinostrobin for the beginning of clinical or toxicological signs. Mortality, if any, was reported over 2 weeks. Then, the animals were sacrificed by giving an overdose of xylazine and ketamine anesthesia on the 15th day. Blood samples were collected by intracardiac puncture and serum was separated for biochemical parameters analysis. Histological and serum biochemical parameters were determined following standard methods (Gwaram et al., 2016, Salga et al., 2017).

### 2.5 Experimental animals for hepatoprotective activity

Sprague Dawley rats were obtained from the Animal House Unit Department of Medical Microbiology, Cihan University-Erbil. Rats weigh between 180 and 200 g were housed individually via wide-mesh wire bottoms to avoid coprophagy throughout the experimental time, at 25˚± 2˚C temperature, approximate moisture 55–65 %, and 12 h’ exposure to light/dark rotation. All the rats were fed on tape water and a standard pellet. The experiment was planned and approved by the Ethics Commission for Animal Research. Human care for whole experimental animals was applied and followed the Guide for Maintenance and usage of laboratory Animals which was produced by the National College of Knowledge and issued through the National Institute of health. Thirty healthy adult male Sprague Dawley rats were arbitrarily alienated into five groups with six rats respectively. Rat’s treatment protocol was determined following the method of (Alkiyumi et al., 2012, Bardi et al., 2014) with a few modifications; Group 1 (normal), which was treated with distilled water (5 mL/kg) i.p. injection for thrice a week, and 10 % Tween 20 (5 mL/kg) via oral administration every day for two months. Group 2 (hepatotoxic) inoculated i.p. (200 mg/kg) of TAA three times a week and daily oral administrated by 10 % Tween 20 (5 mL/kg) for two months. Group 3 (reference drug) was given TAA (200 mg/kg) i.p. injection three times weekly, followed by regular administration of Silymarin (50 mg/kg) for 2 months. Groups 4 and 5 received TAA (200 mg/kg) i.p injection thrice/week for 2 months, and daily oral administration of pinostrobin with 30 mg/kg, (group 4) and 60 mg/kg (group 5) for 2 months, respectively. After the last treatment at the end of the experimental time (two months), all animals were fated for 24 h and then processed for general anesthesia using ketamine and xylazine 30 mg/kg (100 mg/mL), 3 mg/kg (100 mg/mL) (Farghadani et al., 2019). Blood is withdrawn from intracardial puncture and stored in a gel-activated tube for liver functions test (Alshawsh et al., 2011, Omar et al., 2017).

### 2.6 Biochemical parameters (liver function test)

Blood in clot-activator tubes was separated by centrifugation for 15 min at 2500 rpm. A spectrophotometer is used to measure alanine aminotransferase (ALT), aspartate aminotransferase (AST), alkaline phosphatase (ALP), total bilirubin, and total protein in addition to albumin. Biochemical parameters were assessed in the Erbil Hospital Laboratory (Farghadani et al., 2019, Salga et al., 2017).

### 2.7 Macroscopic appearance of liver

A liver assessment was done by opening the rat’s abdominal and thoracic cavities. The livers showed significant macroscopic proof of pathological changes. Also, other organs showed pathological gross lesions but were excluded from the current study. All livers were separately washed in cold saline and checked for any gross pathological abnormalities by taking microscopic images.

### 2.8 Histopathology of liver tissue

#### 2.8.1 Liver tissue staining

Liver samples were washed in cold saline, cut 2 cm cubic, and fixed in 10 % phosphate-buffered formalin. Leica, Germany tissue processor machine was used to process the specimens. and embedded in paraffin. Five µm thickness slices are routinely stained by Hematoxylin &Eosin respectively (Farghadani et al., 2019, Mahmood et al., 2007), and the Masson trichrome stain was used to stain the collagen fibres (Abood et al., 2015). A Nikon microscope was used to evaluate the liver slides for histopathological change and characteristic areas were photographed.

#### 2.8.2 Immunohistochemistry (PCNA) and (α-SMA)

Poly-l-lysine-treated glass slides were used for proliferating cell nuclear antigen (PCNA), and α-smooth muscle actin (α-SMA) staining methods, as previously described in detail (Bardi et al., 2014, Kadir et al., 2014). The propagation directory of PCNA-stained liver slices was determined by counting the proportion of labeled cells per 1000 liver cells, and the number of mitotic cells was expressed as the mitotic index (Abdulaziz Bardi et al., 2013).

### 2.9 Liver tissue homogenates for endogenous (CAT, SOD) enzymes and oxidative stress (MDA)

Neutral ice-cold phosphate buffer saline 10 % (w/v) was used to wash rats’ livers. Teflon homogenizer was used to homogenize liver samples (all steps done on ice), then at 4500 rpm centrifugation for 15 min at 4˚C cell debris was detached. The supernatant was collected to verify antioxidant activity via superoxide dismutase (SOD) and catalase (CAT) analysis kits (Cayman Chemical Company, USA) (Omar et al., 2017). Malondialdehyde (MDA) is a marker of cellular oxidative stress. MDA, assay kit was used to assess the levels of a thiobarbituric acid reactive substance (TBARS, Cayman Company).

### 2.10 Assessment of inflammatory cytokines

Valuation of TNF-α, IL-6, and IL-10 in liver tissue homogenate was accomplished utilizing a marketable ELISA kit from Cusabio Biotech Co., China. Briefly, the liver homogenate was centrifuged at 3000 g for 15 min and the supernatant was used to estimate cytokine levels using a commercial ELISA kit. The assessment was achieved according to the manufacturer’s procedure stated in Rat TNF-α ELISA Kit (109331), Rat IL-6 ELISA Kit (84597), and Rat IL-10 ELISA Kit (84236). Cytokine concentrations were designed using standard purified recombinant cytokines.

### 2.11 Statistical analysis of data

Data analyses were shown as mean ± standard error of the mean (SEM). One-way ANOVA with Tukey post hoc assessment was performed using SPSS software (version 24). The *p*-values statistical meaning at *p* < 0.05.

## 3 Results

### 3.1 Acute toxicity study

**Fig. 1:**
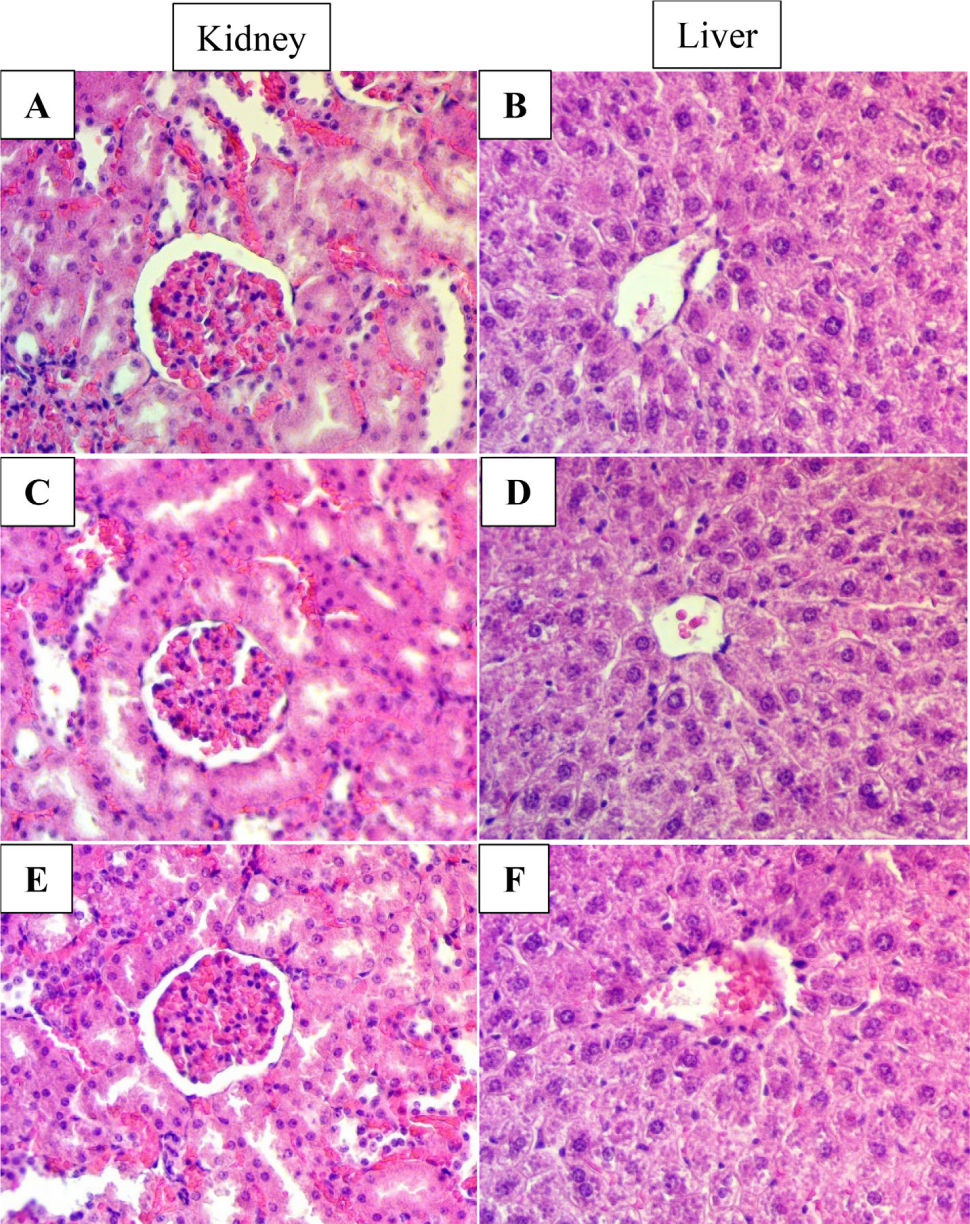
Histological sections of liver and kidney in acute toxicity test. Rats were treated with a 5 mL/kg vehicle (10 % Tween 20) (A and B). Rats were treated with 250 mg/kg (5 mL/kg) pinostrobin (C and D). Rats were treated with 500 g/kg (5 mL/kg) pinostrobin (E and F). No significant changes in the structures of livers and kidneys between the treated and control groups (hematoxylin and eosin stain 40x).

The acute toxicity test did not show any signs of toxicity. There were no histological signs of hepatic and renal toxicity. Moreover, the blood biochemistry parameters examination appeared normal (Fig. 1) (data was not shown and available at request).

### 3.2 Liver biochemical markers

The hepatotoxic effect of TAA was significantly increased (*p < 0*.001) ALT, ALP, total bilirubin, and AST levels indicating liver damage (Table 1). Moreover, the TAA group showed significant decreases (*p <* 0.001, mean ± SE) in total protein and albumin compared with the normal control group, demonstrating acute hepatocellular injury. Pinostrobin and silymarin-treated groups significantly dropped (*p <* 0.001, mean ± SE) enzyme levels of ALT, ALP, total bilirubin, and AST. Furthermore, total protein and total albumin values were elevated (*p <* 0.001) in pinostrobin and silymarin treatments in comparison with the TAA control group. Hence, pinostrobin revoked the hepatotoxic effect of TAA via reinstating typical liver activities. Pinostrobin effectively prevented TAA-induced hepatotoxicity at a dosage of 30 mg/kg, whereas slightly affected it at a dosage of 60 mg/kg.

**Table 1:** The effects of pinostrobin on liver biochemical parameters in rats with TAA-induced hepatotoxicity.

Groups	ALP (IU/L)	ALT (IU/L)	AST (IU/L)	T. Bilirubin (µM/L)	T. Protein (g/L)	T. Albumin (g/L)
Normal Control	70.3 ± 0.6	30.4 ± 0.6	63.9 ± 0.7	1.2 ± 0.01	73.2 ± 0.7	32.9 ± 0.5
TAA + 10 % Tween 20	193 ± 1.1*	130.3 ± 0.5*	172.5 ± 0.5*	5.1 ± 0.07*	45.8 ± 0.6*	13.2 ± 0.2*
Silymarin + TAA (50 mg/kg)	62.4 ± 0.6#	28.1 ± 0.9#	62.5 ± 0.6#	1.4 ± 0.01#	68.1 ± 0.7#	29.4 ± 0.6#
TAA + pinostrobin (30 mg/kg)	58.2 ± 0.8#	22.3 ± 0.6#	55.4 ± 0.5#	1.9 ± 0.03#	60 ± 0.8#	22.1 ± 0.6#
TAA + pinostrobin (60 mg/kg)	54.3 ± 0.5 #	25 ± 0.4#	58.7 ± 0.7#	1.7 ± 0.05#	64.8 ± 0.4#	25.6 ± 0.6#

The effects of pinostrobin or silymarin on serum alanine aminotransferase (ALT), aspartate aminotransferase (AST), and alkaline phosphatase (ALP) activities, as well as total bilirubin, albumin, and protein levels. The data are presented as mean ± SE (n = 6 per group). Significant difference from the normal control group at *p < 0.001, Significant difference from the TAA control group at #*p <* 0.001.

### 3.3 Gross appearance of liver

**Fig. 2:**
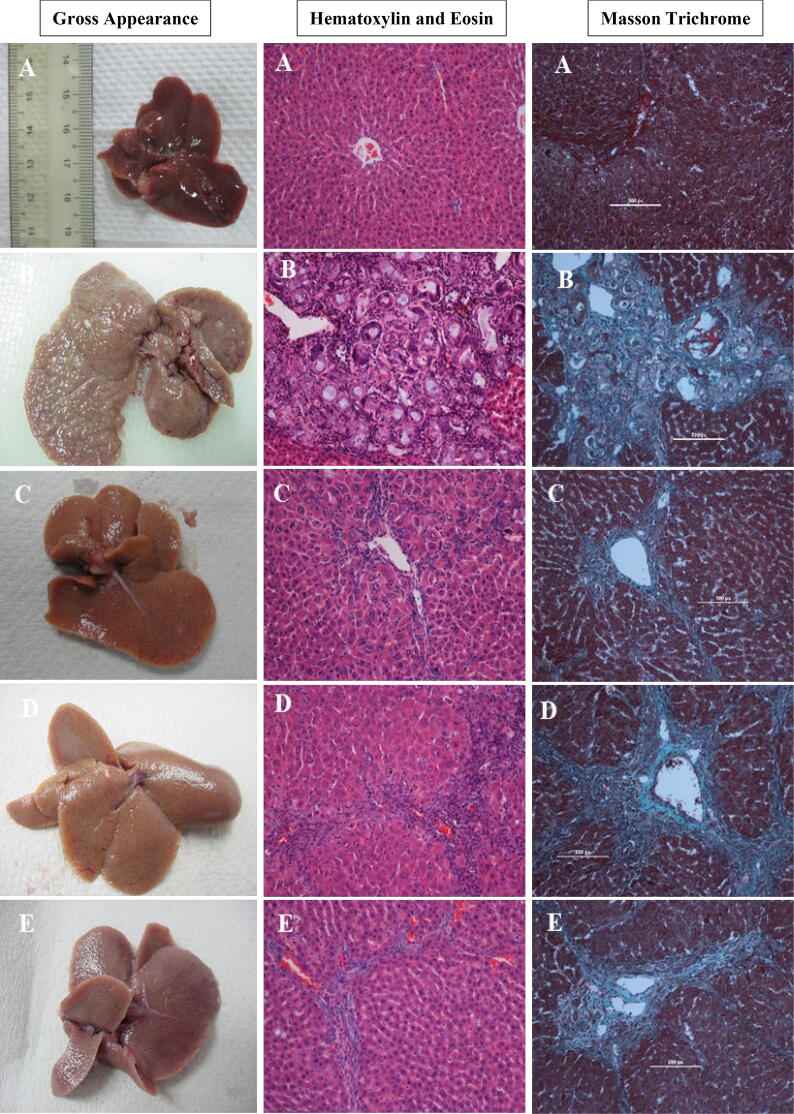
Histopathological examination of liver tissue sections. Hematoxylin and Eosin and Masson Trichrome stains are presenting histopathological sections and Gross appearance of the liver from (A) the normal group, (B) the TAA group, (C) the silymarin group, (D) Pinostrobin low dose group, (E) Pinostrobin high dose group. Stained liver sections were examined under a Nikon microscope (Y-THS, Japan). 20x magnification.

The morphological changes in the liver in all groups (Fig. 2) were evaluated and showed that the normal control group liver had a smooth surface with regular lobs (Fig. 2, A). TAA-induced hepatotoxicity group liver showed an irregular surface with many macros and micro nodules (Fig. 2, B). The TAA + silymarin-treated group had a smooth surface that was similar to the control group (Fig. 2, C). TAA + pinostrobin 30 mg/kg and TAA + pinostrobin 60 mg/kg groups exhibited liver smooth surface and closely maintain the liver’s normal architectural structure and shape (Fig. 2, D, E).

### 3.4 Histopathological examination of hepatocyte sections

Histopathological changes in liver sections stained with hematoxylin and eosin are shown in Fig. 2. Liver slides of the normal group display typical hepatocytes architecture, preserved cytoplasm, and distinguished nucleus and nucleolus with distinct regular plates of liver cells separated by sinusoidal capillaries and central vein (Fig. 2, A). Sections from the TAA group showed irregular hepatocyte architecture resulting from the presence of reforming nodules. Moreover, the liver section was divided via fibrous septa stretching from the central vein to the portal area. Hepatocytes presented severe damage, necrosis and extensive propagation of the bile duct, congested central vein, fatty changes, and granulocytes and monocytes which are presented surround the central vein due to the inflammation (Fig. 2, B). Silymarin + TAA, low and high doses of pinostrobin + TAA groups illustrated relative protection from hepatocyte disruptions induced by TAA. The hepatic cellular compositions showed a reduced amount of damage with a slight fibrotic septum. Insignificant penetration of lymphocytes was observed in these liver section groups. Moreover, the histopathological sections demonstrated remarkable regenerative parenchymal nodules, which are boarded with fibrous tissue as well as noteworthy growth in the cells-fat storing, bile ducts, and Kupffer cells (Fig. 2, C-E).

The liver tissues were stained with Masson's trichrome to assess tissue fibrosis. Collagen deposition was not detected in the normal control liver section (Fig. 2, A). TAA group was shown bile duct regeneration with notable dense fiber septa and increased collagen fiber accumulation around a congested central vein, which is referred to as severe fibrosis in the hepatic tissue (Fig. 2, B). The silymarin, 30 mg/kg, and 60 mg/kg of pinostrobin groups illustrated a reduction in the number of fibrous septa and regeneration nodules. In addition, the collagen fibers in all these three groups were observed to be homologous, which indicated the hepatoprotection activity of pinostrobin extract (Fig. 2, C-E).

### 3.5 Immunohistochemical staining of liver sections

**Fig. 3:**
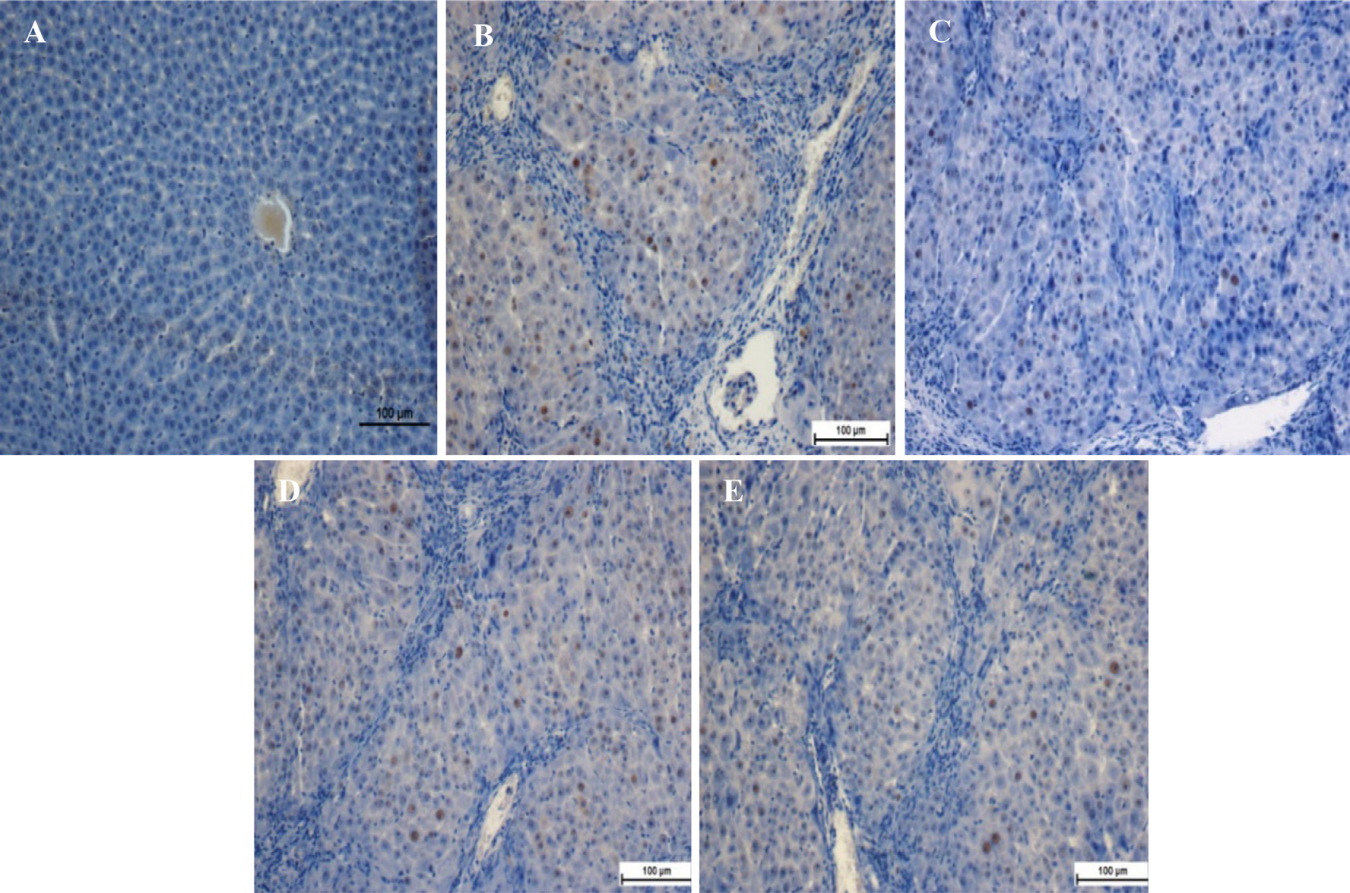
Immunostaining analysis of liver tissue sections. (A) Normal group, (B) TAA group, (C) Silymarin group, (D) Pinostrobin low dose group, and (E) Pinostrobin high dose group. Stained liver sections were examined under a Nikon microscope (Y-THS, Japan). 20X magnification.

The effect of pinostrobin on hepatocyte proliferation after TAA-induced liver injury was observed through immunohistochemical analysis of PCNA appearance in the liver parenchyma using an anti-PCNA antibody (Fig. 3). Hepatocytes in the normal control group showed no PCNA staining, indicating that no cell renewal was taking place. In comparison, hepatocytes from the TAA control group had upregulated PCNA appearance and an increased mitotic directory, showing proliferation to restore the severe liver tissue impairment caused by TAA.

Liver tissues treated with 30 mg/kg pinostrobin, 60 mg/kg pinostrobin, or silymarin had condensed hepatocyte renewal compared to the TAA control group, as designated by abridged PCNA countenance and a significant decrease of the mitotic index. by condensed PCNA appearance and a substantial discount of the mitotic index. Pinostrobin had an excellent outcome on PCNA labeling and mitotic index in a dose-dependent method.

**Fig. 4:**
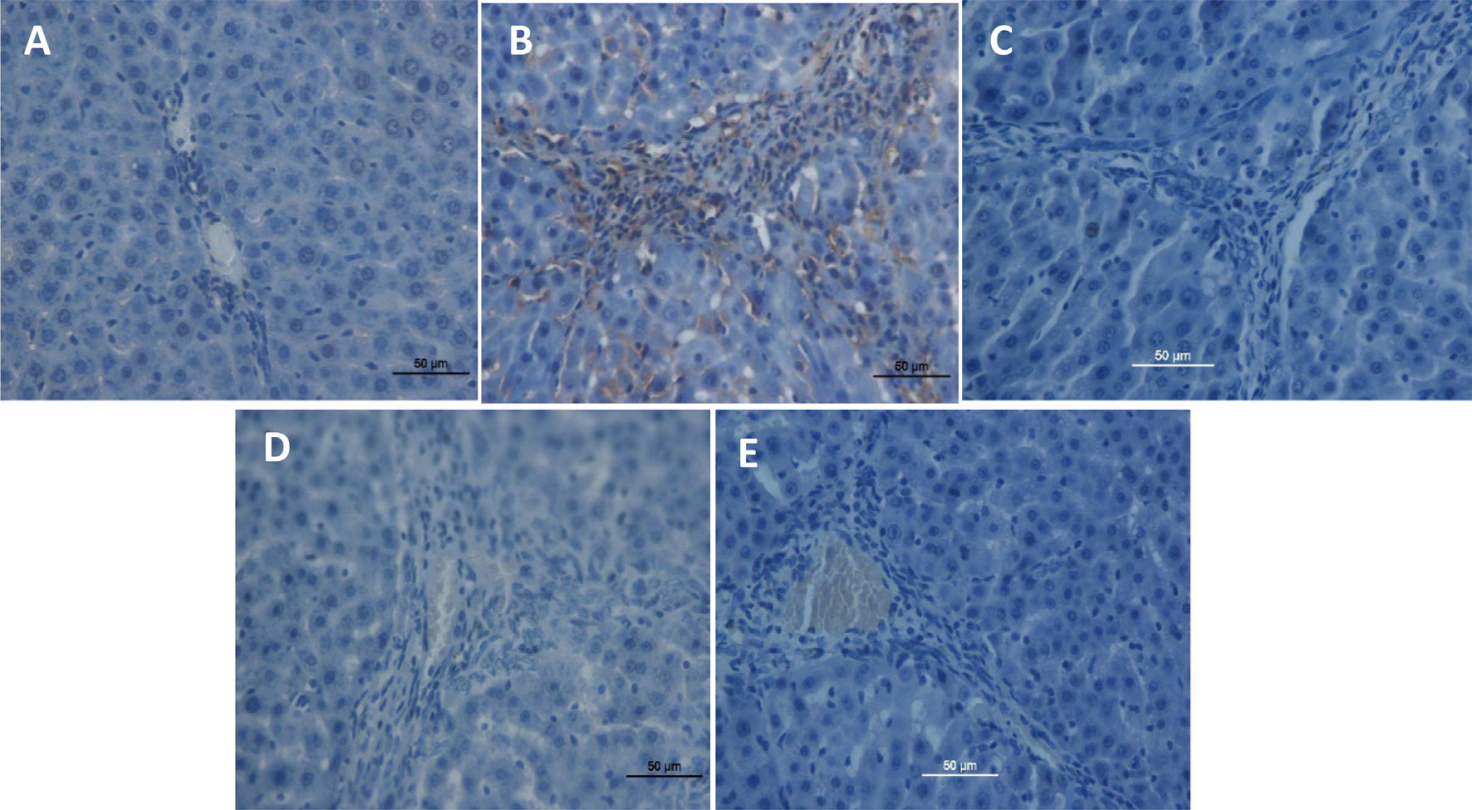
Alpha-smooth muscle actin (α-SMA) in the liver. (A) Normal group, (B) TAA group, (C) Silymarin group, (D) Pinostrobin low dose group, and (E) Pinostrobin high dose group. Stained liver sections were examined under a Nikon microscope with 40x magnification.

TAA-induced liver injury and the importance of pinostrobin were examined by immunohistochemical staining of α-SMA expression in the liver parenchyma utilizing specific antibodies. Down-regulation of α-SMA staining in the normal control group is indicative of the absence of cell regeneration (Fig. 4). On the contrary, the TAA-treated hepatotoxic control group had an outstanding α-SMA appearance signifying up-regulation of these proteins with a higher level of hepatocyte fibrosis. TAA-treated hepatotoxic control group elevated the mitotic figure index significantly suggesting proliferation to the regeneration of widespread hepatic damages induced by TAA. Rat-fed 60 mg/kg pinostrobin had reduced hepatic cell revitalization in comparison to the TAA-treated hepatotoxic control group, as indicated by α-SMA appearance and important lessening of the mitotic index. These results were comparatively similar to that of the silymarin-treated hepatoprotective group. Pinostrobin hepatoprotective treated groups were similar and resisted hepatocyte fibrosis by down-regulating α-SMA expressions. Whereas the 30 mg /kg pinostrobin hepatoprotective group exhibited mild to moderate expressions of α-SMA within the hepatocytes with a significant decrease in mitotic figure index but not analogous to the silymarin-treated hepatoprotective group. These results suggest that pinostrobin hepatoprotective treated groups had an estimable hepatoprotective effect by inhibiting fibrosis of hepatocytes and ameliorating propagation.

### 3.6 Effects of pinostrobin on endogenous antioxidant enzymes in TAA-induced liver cirrhosis in rats

**Fig. 5:**
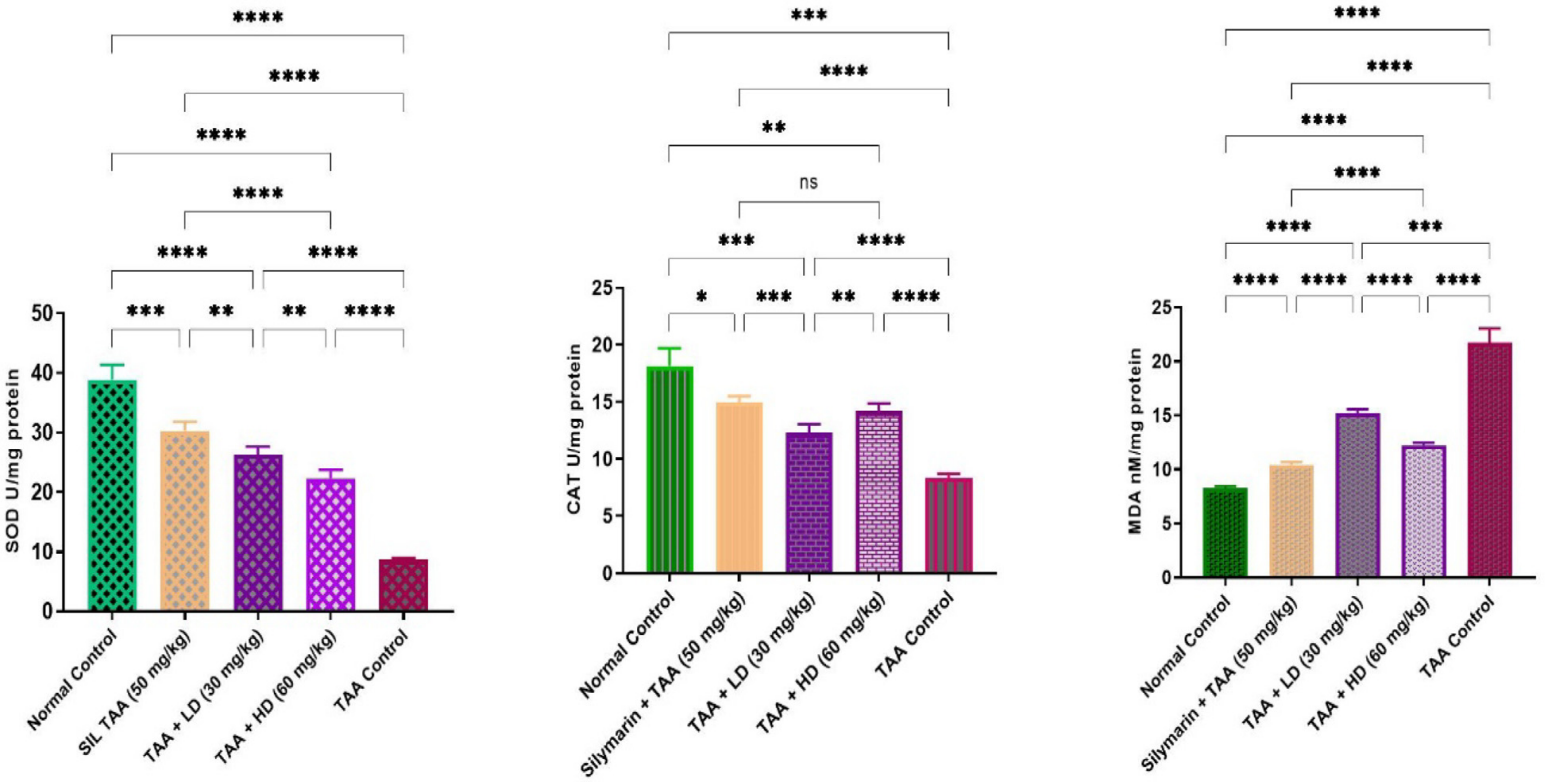
Effects of pinostrobin on antioxidant enzyme activities (SOD and CAT) and MDA level in the liver. Data are expressed as mean ± SEM. Means among groups (n = 6 rate/group) show a significant difference. ns, non-significant; *, *p* < 0.05; **, *p* < 0.01; ***, *p* < 0.001, ****, *p* < 0.0001.

The hepatotoxic group revealed significantly lower SOD and CAT activities, in comparison to the normal group (Fig. 5). Experimental groups fed pinostrobin exhibited significantly restored depletion of SOD and CAT levels to normal values (Fig. 5). The MDA levels were significantly lower in rats treated with pinostrobin when compared to the hepatotoxic control group. The Normal control and silymarin-treated rats showed non-significant changes in their SOD, CAT, and MDA profiles.

### 3.7 Effect of pinostrobin on TNF-α, IL-6, and IL-10 in TAA-induced liver cirrhosis in rats

**Fig. 6:**
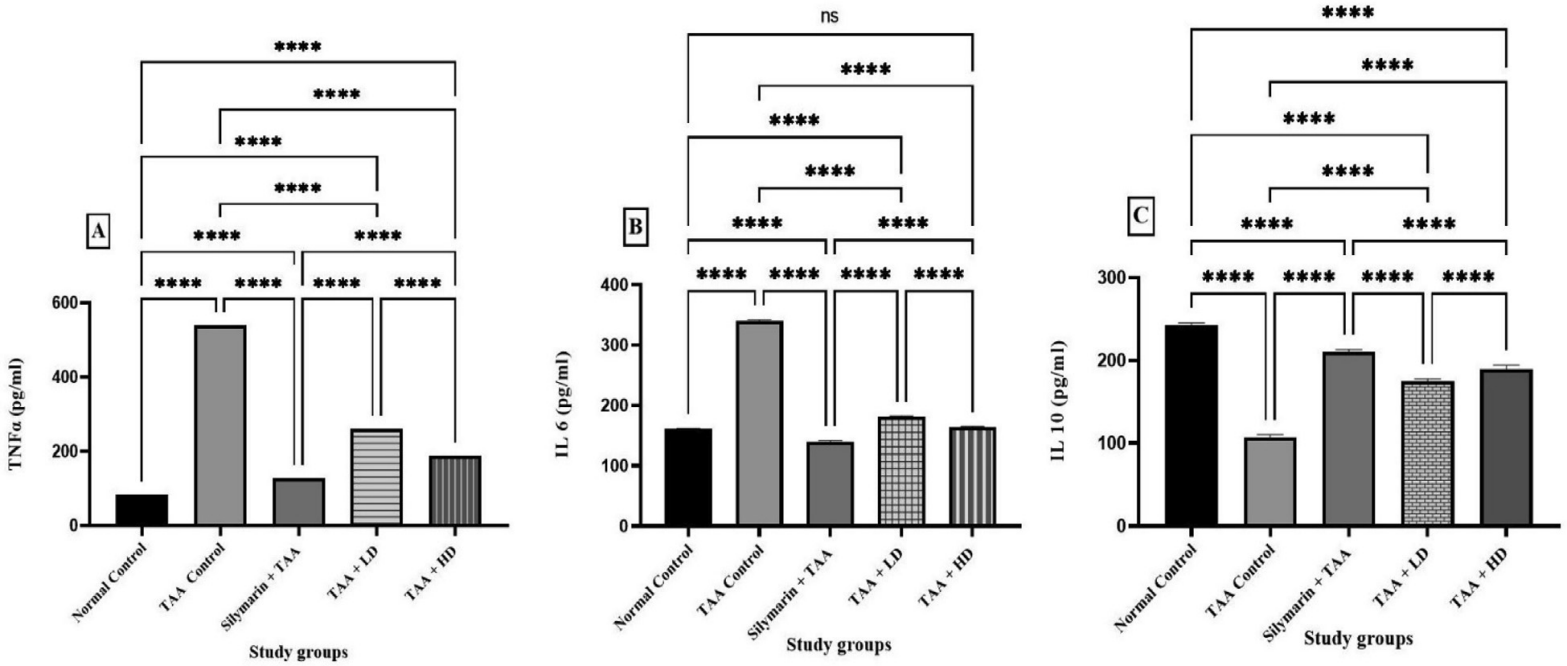
Effect of pinostrobin on TNF-α, IL- 6, and IL-10 on TAA-induced liver cirrhosis in rats. Data are expressed as mean ± SEM. Means among groups (n = 6 rate/group) show a significant difference. ns, non-significant; ****, *p* < 0.0001.

Pinostrobin showed an immune-modulatory influence on liver tissue homogenate by dropping the level of TNF-α and IL-6 and increased in the level of IL-10 (Fig. 6).

## 4 Discussion

The present study was started with an oral acute toxicity trial of pinostrobin on investigational rats, the result revealed safety with no morbidity and death during the whole experimental period even at higher concentrations “i.e., 500 mg/kg of pinostrobin”. Constantly, various investigations by several academics using different medicinal plant extracts or their active ingredients showed safe, and no sign of toxic effect was stated (Salama et al., 2012, Al-Wajeeh et al., 2016, Farghadani et al., 2019, Saremi et al., 2020).

In the present study, the hepatotoxic group was related to a visible increase in activities of liver markers in blood circulation such as ALP, ALT, AST, and bilirubin levels. Similarly, numerous academics reported an increase in liver function markers (Salama et al., 2013, Bardi et al., 2014, Azab and Albasha, 2018). The increase in liver function biomarkers imitates hepatocellular dysfunction. With the consistency of the results of the current study increase in liver markers activities and bilirubin levels in the hepatotoxic group were previously reported by several researchers (Alkiyumi et al., 2012, Salama et al., 2018, Bradosty et al., 2021, Shareef et al., 2022, Al-Medhtiy et al., 2022). These values were meaningfully reduced to near-normal levels after feeding with pinostrobin. With the consistency of our findings, several coworkers used various plant extracts to show reduced liver function enzyme activities and bilirubin levels, which have been previously reported elsewhere (Salama et al., 2018, Abood et al., 2020, Sinaga et al., 2021, Shareef et al., 2022, Al-Medhtiy et al., 2022).

The hepatoprotective achievement may be due to its effect against cell leakage and injury of hepatocyte covering. TAA is specified to burden with RNA initiative from the nucleus to the cytoplasm, starting exterior injury which results in a rising statement of serum liver pointers (Alshawsh et al., 2011, Chen et al., 2018).

In the current study, total protein and albumin quantities in serum were reduced in the TAA control group. Though, silymarin or pinostrobin feeding groups bring back these values to a closely normal level. With the agreement of the results of our investigation enormous numbers of scientists displayed that rats gavaged silymarin or various plant extracts brought the albumin and protein to almost normal levels (Abood et al., 2020, Bradosty et al., 2021, Chavan et al., 2021, Al-Medhtiy et al., 2022) reported that ethanolic leave extracts of *Garuga pinnata* can serve as promising herbal medicine for the treatment of both acute and chronic hepatotoxicity due to the presence of flavonoids.

Outcomes of the existing research showed a decline in collagen deposition in pinostrobin-fed groups in tissue sections stained with Masson's trichrome dye. Similar to the findings of the present investigation, other researchers used a variety of plant extracts to support the finding that collagen fibers were reduced when compared to the TAA control group (Abdulaziz Bardi et al., 2013, Kadir et al., 2014, El-Baz et al., 2019, Abood et al., 2020).

Histopathological (H & E staining) and Masson’s Trichrome staining, and immunostaining displayed the repressing effect of feeding with pinostrobin, which could be owing to its capability to prevent hepatocyte propagation, as designated by down-regulation of PCNA staining. Similarly, exposure that green tea potentially inhibited the progression of liver cirrhosis, and down-regulation of PCNA proliferation (Shareef et al., 2022). The results of the existing study exhibited that the normal liver group or silymarin-treated collections demonstrated down-regulation of PCNA, suggesting the absence of cell regeneration. Up-regulation of PCNA countenance hepatocytes was observed in a hepatotoxic set, exemplifying comprehensive construction, and imaginable exertion to the reconstruction of tissue impairment (El-Lakkany et al., 2019, El-maadawy et al., 2021). Otherwise, rats fed with silymarin or pinostrobin dramatically reduced cell proliferation PCNA stain. In scientific literature, huge numbers of remedial plants with hepatoprotective potential have been noticeable by other investigators (Giribabu et al., 2018, Marzouki et al., 2019, Keshk et al., 2019, Abd Eldaim et al., 2021).

In TAA treated hepatotoxic group, TAA formed reactive-oxygen-species (ROS) producing activation of hepatic satellite cells (HSC) which is the main source of extracellular matrix (ECM) manufacture in chronic liver cirrhosis and up-regulation of α-SMA. Stimulation of HSC is accompanied by cell propagation and upgrading of ECM construction, the appearance of α-SMA to myofibroblasts (Kadir et al., 2014). The marks of our research accessible pinostrobin feeding down-regulated appearance of α-SMA compared to the hepatotoxic group which exhibited noticeable up-regulation of α-SMA. Pinostrobin significantly prevents HSC activation by avoiding the creation of ROS. Several types of research by various researchers found the down-regulation of α-SMA in TAA-induced liver cirrhosis (Yang et al., 2019, Ujiie et al., 2020).

In the present study, SOD, and CAT, in liver tissues homogenate significantly decline in the hepatotoxic group as compared to the normal group. Both enzymes become flagged by free radicals resulting in liver weakening (Gowifel et al., 2020). Meanwhile, pinostrobin expressively elevated the concentration of serum CAT and SOD by the self-protective liver from the injurious influence of free radicals compared to the TAA control group. Matching outcomes have been described formerly by uncountable researchers (Salama et al., 2018, Shareef et al., 2022, Al-Medhtiy et al., 2022). MDA as a lipid peroxidation marker is a usual injurious process (El-Mihi et al., 2017, Gowifel et al., 2020). MDA levels elevated in tissue improved lipid peroxidation (Hajrezaie et al., 2015). Rise MDA initiates damages and tragedy of antioxidant protection to block the expansion of additional free radicals (Zheng et al., 2018). The existing search exhibited TAA yield increase in MDA quantity has been promisingly reduced by pinostrobin feeding. Parallel results have been previously reported by various academics elsewhere (Salama et al., 2018, Zaidi and Mahboob, 2020, Bradosty et al., 2021). A drop in hepatic SOD and CAT activities in the hepatotoxic group might explain elevated MDA.TAA created liver fibrosis in rats. Nonetheless, a rat gavaged with pinostrobin could dramatically accelerate the recovery of the liver injuries suggestively preventing the impact of TAA intoxication. These results are likewise consistent with former studies stated by abundant inventors using diverse medicinal plants (Rouhollahi et al., 2015, Salama et al., 2018, Said et al., 2019, Abood et al., 2020). Shareef *et al.* showed that green tea potentially inhibited the progression of liver cirrhosis, prevented oxidation of hepatocytes, recovered SOD and CAT enzymes, condensed MDA, and reduced cellular inflammation (Shareef et al., 2022).

TAA induces an inflammatory response that initiates a dynamic chain of immune responses associated with the release of vast amounts of inflammatory cytokines such as TNF-α and IL-6, which in turn produce an increased quantity of ROS (Wei et al., 2003). TNF-α, being a major proinflammatory cytokine produced by macrophages, attracts neutrophils to liver injury (Martin and Wallace, 2006, Kishimoto, 2005). IL-6 is another important proinflammatory cytokine shown to mediate immune response and acute inflammation. IL-6 activates granulocytes and agranulocytes, which in turn trigger a stress response in injured tissue (Mei et al., 2012, Sabat et al., 2010) suggesting that IL-10 can suppress inflammatory response and inhibit TNF-α production. Previous reports showed that TAA was able to increase proinflammatory cytokines and decrease anti-inflammatory cytokines in liver tissue (Jadaun et al., 2019, El-Lakkany et al., 2019). Our results were in agreement with these observations, where exposure to TAA showed elevated TNF-α and IL-6 and a decrease in IL-10 levels, compared to normal controls. However, pretreatment inhibited the depletion of IL-10 and elevation of TNF-α and IL-6 levels, which shows its anti-inflammatory effect on TAA-induced liver cirrhosis in the rat.

## 5 Conclusion

The current study found that pinostrobin had a significant hepatoprotective effect in reducing TAA toxicity in rats, as evidenced by biochemical liver parameters, endogenous enzymes, histology, and immunohistochemistry. Pinostrobin intensely raises the CAT & SOD activities, with a significant reduction of hepatic MDA. The hepatoprotective effect of pinostrobin could be attributed to its ability to inhibit hepatocyte multiplication, reduce oxidative stress and lipid peroxidation, down-regulate PCNA, and α-SMA, possess antioxidant and free radical scavenger properties, and modulating proinflammatory cytokine.

## 6 Ethics announcement

1. The current experiment was authorized through the conscience team for animal investigation, Faculty of Science, Cihan University-Erbil, and Ethic No. ERB, 115, 11/03/2019. All animals for the duration of trials, obtained human attention by principles set forth by the “Director for the Maintenance and Use of research laboratory Animals” which was organized by the Nationwide School of Sciences has issued by the National Institution of healthiness.
2. This manuscript has not been published in whole or in part elsewhere.
3. The manuscript is not currently being considered for publication in another journal.
4. All authors have read and approved the manuscript.

## CRediT authorship contribution statement

**Suhayla Hamad Shareef:** Conceptualization, Data curation, Formal analysis, Investigation, Methodology, Software, Supervision, Visualization, Writing – original draft, Writing – review & editing. **Morteta H. Al-Medhtiy:** Investigation, Writing – review & editing. **Ahmed S. Al Rashdi:** Investigation, Funding acquisition. **Peshawa Y. Aziz:** Visualization, Writing – review & editing. **Mahmood A. Abdulla:** Conceptualization, Data curation, Investigation, Methodology, Supervision, Writing – original draft, Writing – review & editing.

## Declaration of Competing Interest

The authors declare that they have no known competing financial interests or personal relationships that could have appeared to influence the work reported in this paper.
